# Nanobody Engineering: Toward Next Generation Immunotherapies and Immunoimaging of Cancer

**DOI:** 10.3390/antib8010013

**Published:** 2019-01-21

**Authors:** Timothée Chanier, Patrick Chames

**Affiliations:** Aix Marseille University, CNRS, INSERM, Institute Paoli-Calmettes, CRCM, 13009 Marseille, France; timothee.chanier@inserm.fr

**Keywords:** Nanobody, Single Domain Antibody, Cancer, Immunotherapy, Imaging

## Abstract

In the last decade, cancer immunotherapies have produced impressive therapeutic results. However, the potency of immunotherapy is tightly linked to immune cell infiltration within the tumor and varies from patient to patient. Thus, it is becoming increasingly important to monitor and modulate the tumor immune infiltrate for an efficient diagnosis and therapy. Various bispecific approaches are being developed to favor immune cell infiltration through specific tumor targeting. The discovery of antibodies devoid of light chains in camelids has spurred the development of single domain antibodies (also called VHH or nanobody), allowing for an increased diversity of multispecific and/or multivalent formats of relatively small sizes endowed with high tissue penetration. The small size of nanobodies is also an asset leading to high contrasts for non-invasive imaging. The approval of the first therapeutic nanobody directed against the von Willebrand factor for the treatment of acquired thrombotic thrombocypenic purpura (Caplacizumab, Ablynx), is expected to bolster the rise of these innovative molecules. In this review, we discuss the latest advances in the development of nanobodies and nanobody-derived molecules for use in cancer immunotherapy and immunoimaging.

## 1. Introduction

It is now well established that tumor cells can interact with their environment to promote an immunosuppressive environment to favor their survival and proliferation. Targeting the tumor environment for therapy has become a major interest in the past decade and is now a paradigm for new cancer therapies. Success of immunotherapy in cancer treatment, particularly the use of PD-1/PD-L1 and CTLA-4 antibodies, has led to the development of treatment targeting other immunological pathways [1,2]. However, immunotherapies are only efficient in a fraction of cancer patients [3]. Combination therapies are emerging as the path to increase response rates and tackle cancer cell escape mechanisms [4]. Their success often relies on the presence of immune cell within the tumor and their interaction with immunosuppressive ligands expressed by tumor cells. Cancers are currently best classified according to the immune infiltrate as well as the tumor cell type and localization [5].

In the case of non-infiltrated (“cold”) tumors resistant to checkpoint inhibitors, new immunotherapy approaches tend to use bispecific construction targeting a tumor antigen and an immune receptor to favor immune cells infiltration and tumor cell specific targeting. Two bispecific antibodies have been approved by the US food and drug administration (FDA) (catumaxomab, CD3 × EpCAM and blinatumomab, CD3 × CD19) and many more are under clinical or pre-clinical development [6]. With the rise of molecular antibody engineering, a lot of different bispecific formats combining the heavy and light variable domains (VH + VL) with different specificities are being used for various therapeutic modalities [7].

**Figure 1:**
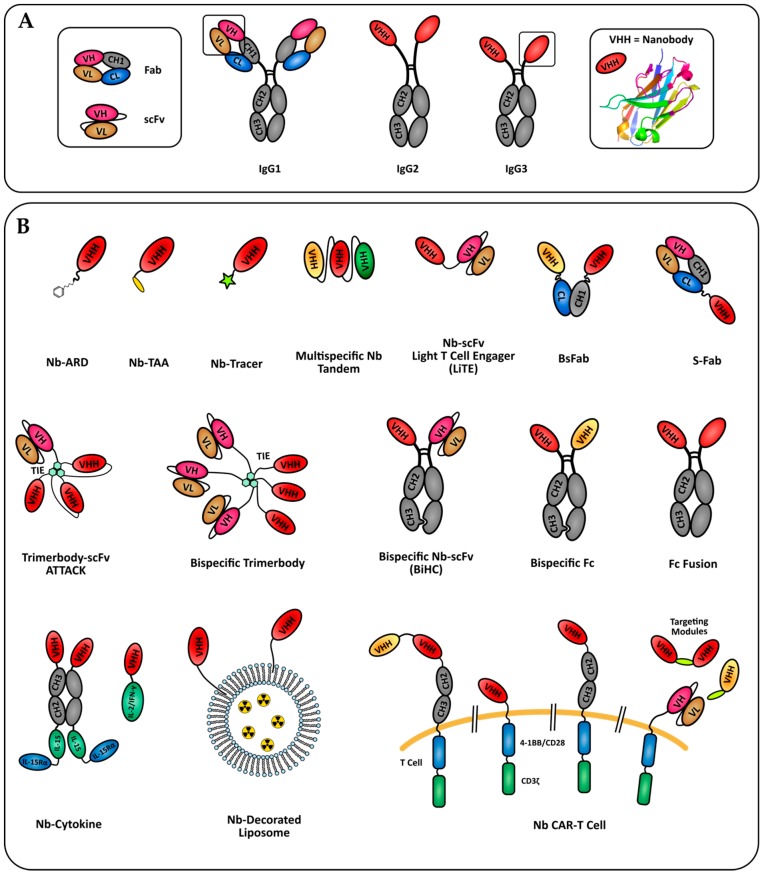
Nanobody-based formats in development for tumor immunotherapy and imaging. (**A**) Camelids specificity domains derived of conventional IgG1 or HcAbs (IgG2 and IgG3). The nanobody crystal structure shown is pdb entry 6GZP. (**B**) Formats of nanobody engineered molecules discussed in this review. Nb: nanobody; ARD: antigen recognition domain; TAA: tumor associated antigen.

**Figure 2:**
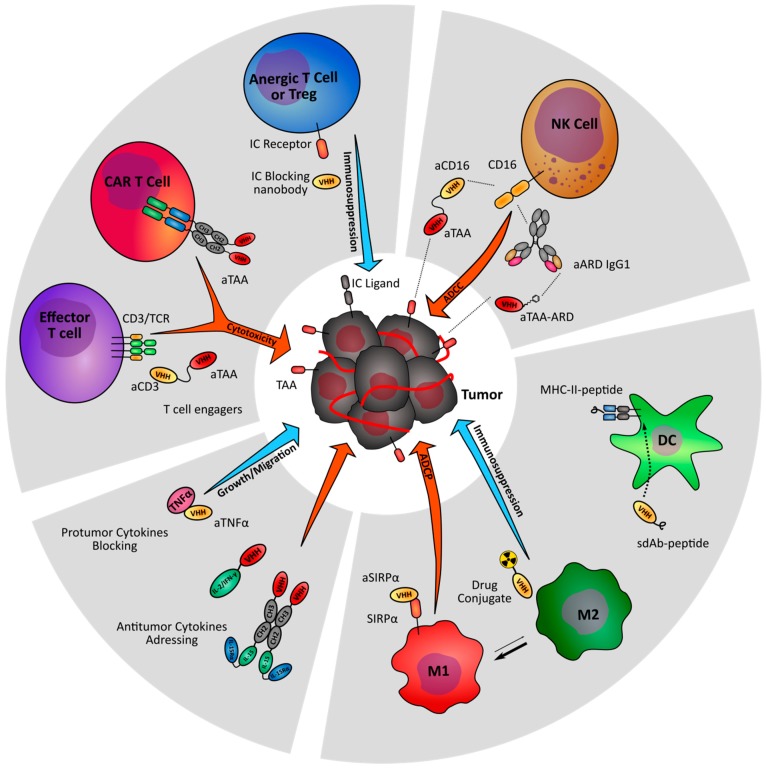
Nanobody-based strategies targeting the immune stroma of tumors. Nanobody-derived immunomodulatory molecules are under investigation to increase anti-tumor immunity (orange arrows) and prevent tumor-driven immune suppression (blue arrows). TAA: Tumor associated antigen; IC: Immune checkpoint; ARD: Antibody recruiting domain.

Heavy chain only antibodies (HcAbs) have been identified in camelids. These antibodies are lacking the CH1 domain compared to conventional IgGs and are devoid of light chain. The specificity of HcAbs only relies on heavy variable domains called VHH. The recombinant production of a VHH generates a fragment called single domain antibody (sdAb), or nanobody [8]. Thanks to their high degree of sequence identity with human VHs (of family 3), nanobodies are expected to exhibit a low immunogenicity in human, and are easy to humanize for therapeutic perspectives [9], as confirmed by several phase clinical trials involving nanobodies and the recent approval by the European medicines agency (EMA) of the first therapeutic nanobody, caplacizumab [10]. The CDR3 loop of nanobodies is usually longer than conventional VH, allowing the binding to non-conventional epitopes such as protein clefts [11]. Moreover, structural studies have established that nanobodies usually have greater paratope diversity, involving amino acids within variable loops and framework regions [12]. Nanobodies are also characterized by a good solubility and stability to pH and temperatures. Importantly their small size allows for a better penetration within tissue and in cell–cell interfaces like immune synapses [13]. Conversely, this can be seen as a disadvantage for therapy, due to a quick renal elimination causing a very short serum half-life (close to 30 min). Different strategies to increase their serum half-life have been developed. One of them is based on a fusion to anti-albumin nanobody, increasing the serum half-life to 4–10 days without drastically increasing the molecule size [14]. Other strategies consist in a fusion to a human Fc fragment (CH2 and CH3 domains) allowing neonatal Fc receptor-based recycling and generating a bivalent molecule with a higher apparent affinity. Nanobodies can also be used to engineer larger molecules with several valencies or specificities and can be easily conjugated to imaging agent or drug delivery systems. Importantly, their high modularity increases further the format possibilities to crease small size antibody-derived molecules for therapy and imaging (Figure 1). In this review, we discuss the potential of nanobodies and nanobody-based engineered molecules for the immunotherapy and immunoimaging of tumors (Figure 2).

## 2. Targeting T Cell Activation and Cytotoxicity

T cells are adaptive immune cells endowed with a crucial role in anti-tumor cytotoxicity. Following the success of immune checkpoint blockade therapies, different strategies based on monoclonal antibodies aiming at activating the T cell mediated anti-tumor response and overcome immunotherapy resistance are currently under intense investigation [15]. Within this line, the high modularity of nanobodies is being actively exploited to design innovating drug candidates. Current research mostly covers T cell redirection strategies through the generation of bispecific format to recruit and activate cytotoxic or γδ T cells, and the design of nanobody-derived chimeric antigen receptor (CAR) T cells. Some immune checkpoint blocking nanobodies are also being developed.

### 2.1. CD3 Bispecific Nanobodies: BiTE-Like Formats

CD3, and most specifically its ζ chain, belongs to the T cell receptor (TCR) complex. Anti-CD3 antibodies are commonly used to activate T cells in vitro. However therapeutic anti-CD3 antibodies can induce systemic inflammation and strong adverse effects. Bispecific antibodies allow a specific recognition and killing of tumor cells by bridging together the tumor and immune cells. Importantly, monovalent CD3 binding in the periphery does not trigger T cell activation and cytokine release syndrome. Bispecific T cell engagers (BiTEs) rely on a bispecific format combining an anti-CD3 single chain Fv (scFv) with a tumor associated antigen (TAA) specific scFv. BiTEs have been developed against several tumor antigens and blinatumomab (Blincyto, Abgen), a CD3 × CD19 BiTE is now approved by the FDA for the treatment of B-cell acute lymphoblastic leukemia [16]. Nanobodies allow for generation of BiTE-like format with a smaller size and higher modularity, and have been used to target several tumor antigens.

Her2 is a member of the epidermal growth factor receptor (EGFR) family, commonly known as a tumor antigen over-expressed in 20 to 30% of breast cancers, but also expressed in many other cancer types [17]. An anti-Her2 antibody-based immunotherapy with two monoclonal antibodies targeting different Her2 epitopes (trastuzumab + pertuzumab) is now first line treatment for patients with metastatic Her2^+^ breast cancer. Lin et al. designed the S-Fab format, combining the Fab fragment of the well characterized anti-CD3 UTCH1 clone bearing an anti-Her2 nanobody fused to the C-terminus of its light chain [18]. The construction led to encouraging pre-clinical results, including a strong in vitro cytotoxicity and in vivo tumor growth inhibition. An alternative of the CH1-Cκ heterodimerization motif is the knob-into-hole technology, relying on CH3 engineering to prevent homodimerization of two distinct CH3 mutants [19]. The resulting bispecific Fc fragment increases the size of the molecule to ~80 kDa, still significantly smaller than a full-size IgG (~150 kDa). This format retains the serum half-life and effector functions of an IgG while affording a higher tissue penetration. A Her2 × CD3 bispecific antibody using the anti-CD3 UTCH1 scFv and an anti-Her2 nanobody (Nb) was generated with this technology [20]. The construction led to a strong anti-tumor growth effect with 4 doses spaced every 3 days instead of daily doses for 7 days for the previous Fab-Nb format.

Carcinoembryonic antigen (CEA) is a cell surface GPI-anchored glycoprotein commonly overexpressed in solid tumors [21]. It is a popular antigen for targeted therapy designs. A CEA × CD3 S-Fab showed strong T cell-mediated cytotoxicity against CEA^+^ colon carcinoma cells LS174T and in vivo tumor inhibition [22]. In a more recent study, the authors used PEGylation to increase the in vivo serum half-life of the construct from 3 to 36 h in rats [23]. The anti-tumor effect was not impacted in a daily injection model, showing that PEGylation does not reduce the molecule activity, thus permitting to reach the same efficacy with less frequent injections.

Mølgaard et al. generated a bispecific light T cell engager (LiTE) comprising an anti-CD3 UTCH1 scFv fused in N- or C-terminal with an anti-EGFR nanobody [24]. The very small size of the LiTE format leads to a high tissue penetration but requires continuous injection for therapeutic applications or gene-based delivery. The authors reported similar binding properties and in vitro effects for C and N terminal fusions. The team used the nanobody modularity to build an original new format combining the CD3 scFv and 3 anti-EGFR nanobodies in a trimerbody format [25]. The interest of this format resides in a controlled orientation of the 3 nanobodies via collagen-derived trimerization domains (TIE), causing an increased affinity for EGFR without losing affinity for CD3. Although high affinity to tumor antigens can also lead to off-target effect on healthy cells expressing the receptor at lower levels, this model can be applied to target more tumor-specific antigens or to combine several lower affinity nanobodies. This format was further developed into a bispecific trivalent 4-1BB × EGFR construct combining 3 4-1BB scFv-TIE-EGFR Nb chains [26]. The 4-1BB is a strong co-stimulatory receptor expressed on T and NK cells and induces cytotoxicity, proliferation and cytokine release. However, systemic activation of 4-1BB causes over-activation of T and NK cell responses and sometimes severe toxicities. This trivalent/bispecific format was able to mainly localize into tumors, allowing an efficient anti-tumor effect with significantly reduced off-target cytotoxicity.

### 2.2. γδT Cell Activation

γδT cells are a subpopulation representing 1–10% of leukocytes. Their activation is MHC-independent, and leads to cytokine release and cytotoxicity. They are a heterogeneous population with pro-tumor (regulatory γδT, γδT17) and anti-tumor (Vγ9Vδ2 T) activities. Vγ9Vδ2 T cells constitute the main population of γδT cells and their tumor infiltration have been associated with good prognosis factor in various tumor types [27,28,29]. Proliferation and activation of anti-tumor Vγ9Vδ2 T cells appear as a promising therapeutic strategy [30]. De Bruin et al. screened several nanobodies for their ability to specifically bind to Vγ9Vδ2 TCR and activate these γδ T cells [31]. Using one of the activating nanobodies, they built a bispecific antibody combining anti-EGFR and anti-Vγ9Vδ2 TCR nanobodies, separated with a G_4_S linker [32]. This antibody was able to activate Vγ9Vδ2 T cells and triggered in vitro cytotoxicity against EGFR^+^ cancer cell lines. In humanized xenografted mice, Vγ9Vδ2 T cells injection alone caused improved survival and lower tumor burden and this effect was further increased with co-injection with the bispecific antibody.

### 2.3. Engineering Nanobody-Derived TCR in CAR-T Cell Therapy

Alternatively, T cell retargeting can be achieved through genetic engineering. Chimeric antigen receptor (CAR) T cells are genetically modified T cell expressing a non MHC-restricted antigen receptor. The intracellular domain is a fusion between a CD3ζ signaling domain and one or two co-stimulatory intracellular domains (CD28, OX-40, 4-1BB), and the extracellular domain is an antigen-binding site derived from an antibody. Great success and FDA-approval of anti-CD19 scFv-based CAR T cells (KYMRIAH) for treatment of B cell ALL has further increased interest in this field for cancer therapies. The main issues with CAR T cells are adverse reactions and the difficulty to control T cell proliferation in patients [33]. Many CAR T cell formats have been described, varying substantially is their antigen binding site, extracellular spacer and intracellular signaling domains.

More recently, CARs have been designed with a nanobody as antigen recognition site. The first nanobody-based CAR T cell used a MUC-1 targeting nanobody [34]. Interestingly, in a second generation of this CAR T cell, the authors added a caspase8-mediated suicide switch to control in vivo T cell proliferation and reduce potential unwanted damages [35]. This switch is triggered by adding a dimerization protein inducing caspase8 cell death signaling.

A novel modular universal CAR platform technology named UniCAR was developed to control the T cell activity and proliferation after injection. The authors used an inert CAR directed against a short peptide (5B9) and a targeting module (TM) directed against a tumor antigen and tagged with 5B9 [36]. The specific anti-tumor activity of these CAR T cells relies on the presence of a targeting module that can be switched during treatment to change antigen specificity, or to stop treatment. Recently, a nanobody-based anti-EGFR CAR-T cell was generated using the UniCAR technology [37]. As expected, these CAR T cells were unable to induce significant lysis of EGFR^+^ cells and cytokine release in the absence of the anti-EGFR TM. PET imaging showed that the TM was rapidly eliminated via kidneys and that TM-CAR complexes were reversible. The team recently published a nanobody-based bivalent anti-EGFR module with increased affinity for EGFR to be used in the UniCAR system [38]. The increased affinity allows binding on EGFR^low^ cells. The bivalent construct showed increase cytokine release and tumor rejection. This strategy could also be used to produce bispecific CAR T cells.

Bispecific CAR T cells can be designed to reduce the risk of tumor escape by loss of a tumor antigen and increase targeting accuracy. A bispecific Her2 × CD20 CAR was designed using a dual nanobody tandem as antigen recognition domain [39]. In this study, the dual specific CAR were equally efficient against CD20 or Her2 cells, proving the potential of bispecific CAR T cells to overcome the loss of antigen issue.

Sharifzadeh et al. generated different CAR formats with an anti-TAG72 nanobody to compare different costimulatory domains. They confirmed that CD28-OX40-CD3ζ CAR was more potent than the shorter CD28-CD3ζ intracellular domain. Similarly, significant anti-tumor effects in mice was achieved with nanobody-derived CAR targeting Her2 in breast cancers [40], glypycan-2 in neuroblastoma models [41] or CD38 in multiple myeloma [42].

### 2.4. Immune Checkpoint Blockade

Immune checkpoints are a group of inhibitory receptors mainly expressed in T and NK cells. The success of checkpoint therapy led to FDA approval of monoclonal antibodies against PD-1, PD-L1, and CTLA-4 [43]. Nanobodies are being developed as an alternative to monoclonal antibodies for therapy or as tools to investigate immune checkpoint biology. For instance, using an anti-murine CTLA-4 nanobody either as a monovalent domain, as a bivalent dimer or fused to an IgG2a constant region, Ingram et al. demonstrated that blocking CTLA-4 was not sufficient to trigger an anti-tumor activity. Indeed, only a Fc fusion of the nanobody triggered Treg depletion, controlled tumor growth and increased overall survival, suggesting a role of the Fc receptor-mediated immune response through the involvement of NK-mediated antibody dependent cell-mediated cytotoxicity (ADCC) [44]. However, another study showed an anti-tumor effect for a monovalent anti-CTLA-4 nanobody in a similar B16 mice model [45].

A recent study demonstrated a comparable level of CD4^+^ T cell activation and tumor growth inhibition for a bivalent anti-PD-L1 nanobody-Fc fusion and durvalumab (a FDA-approved anti-PD-L1 IgG1κ) [46]. In addition to T cell activation, an effect was mediated by ADCC against PD-L1^+^ tumor cells, consistent with the known involvement of FcγRs in PD-1/PD-L1 therapies [47].

Other checkpoint blocking nanobodies are being investigated, such as an anti-TIM-3 nanobody that demonstrated an anti-proliferative effect against the human leukemia cell line HL-60 [48].

## 3. Enhancing NK Cell-Mediated Antitumor Activity

### 3.1. Anti-CD16 Bispecific Antibodies

NK cells are innate effector immune cells playing a role in eliminating malignant cells and pathogens. They play a role in early cancer killing and immunosurveillance [49]. NK cell infiltration within tumor is a good prognosis in cancer patients [50]. They mediate direct killing of tumor cells independently of antigen presentation. NK cells are also important mediators of the adaptive immune response through cytokines and chemokine release, dendritic cells recruitment and T cell activation [51,52,53].

CD16 (FcγRIII) is a receptor for IgG1 and IgG3 Fc fragment expressed on NK cells, macrophages and γδT cells and mediates ADCC and phagocytosis of antibody-opsonized cells. NK cell-mediated ADCC is an important factor in the success of anti-tumor antigens antibody therapies and CD16 polymorphisms with higher affinity for Fc fragment are correlated with improved response to these treatments [54,55,56,57]. Fc engineering has become a major approach to improve efficiency of therapeutic antibodies [58]. As stated previously, the presence or absence of an Fc fragment can impact the action of nanobody-based constructs [44]. The engagement of CD16 also activates NK cell proliferation and functions independently of the ADCC effect through PI3K/MAPK pathways, without the need of a costimulatory signal [59,60]. The use of CD16 × TAA bispecific antibodies rather than Fc fragments was proposed to avoid binding to other Fc receptors, and sensitivity to CD16 polymorphisms. C21, A nanobody with high affinity for CD16 (10 nM, as determined by Biacore) was able to induce IL-2 and IFNγ secretion by NK cells in vitro after multimerization by sdAb biotinylation and incubation with streptavidin [61]. Different bispecific formats using the C21 nanobody are being developed to achieve this dual effect to improve tumor targeted therapy and allow a better recruitment of NK cells.

*Tandem*: The smallest bispecific nanobody format consists in a tandem of two nanobodies linked head to tail by a short peptide linker. Wang et al. constructed two tandems based on the anti-CD16 nanobody C21 and an anti-MUC-1 nanobody with a 2 amino acid linker (GS) [62] or an anti-CEA nanobody with a larger linker ((G_4_S)_3_) [63]. The major interests of this format are an effective and low-cost production in bacterial systems and a high stability compared to classical scFvs. In both studies, the authors observed a high in vitro cytotoxicity and in vivo tumor growth inhibition in NOD/SCID mice xenografted with MUC-1^+^/CEA^+^ colon carcinoma line LS174T, humanized by PBMC injection and treated daily with bispecific antibody injections. Therapeutic molecules based on this format would thus require PEGylation or the addition of an anti-albumin binding domain to increase their serum half-life, or the use of infusion pumps to deliver a constant flow rate, such as those used for blinatumomab in the clinic.

*BsFab*: The so-called bsFab format, combining the C21 nanobody with an anti-CEA nanobody through CH1-Cκ heterodimerization motif was developed [64]. The team showed that albeit the bsFab alone could not activate CD16-transfected Jurkat cells, it could induce IL-2 and IFN-γ secretion through CD16 clustering in the presence of CEA^+^ colon carcinoma cells (LS174T). The bsFab showed potent in vitro NK cytotoxicity against CEA^+^ cancer cells independently of CD16 polymorphisms, leading to tumor growth inhibition in vivo. Such bispecific formats might constitute a promising approach to specifically activate NK cells within tumor tissues. A similar construct using C21 nanobody with an anti-Her2 nanobody was compared to Trastuzumab [65]. The anti-Her2 bsFab showed increased cytokine release in vitro and similar tumor growth inhibition in vivo on Her2^+^ breast cancer cell lines SK-BR-3 and BT 474. Interestingly the bsFab also led to NK cell activating and tumor growth inhibition using the trastuzumab-refractive Her2^low^ cell line MCF-7 model.

*BsFc*: A bispecific CD16 × CEA was generated using anti-CD16 and anti-CEA nanobodies linked to two different mutants of an IgG1 to produce a bispecific Fc via the knob-into-hole technology [66]. This construct resulted in tumor growth inhibition in mice models. Unfortunately, the half-life of this molecule was not assessed in this study but can be expected to be higher than bsFab or tandem formats.

*S-Fab*: Wang et al. used the S-Fab format by combining an anti-CD16 Nanobody with the Fab of the anti-Her2 mAb Trastuzumab [67] or Pertuzumab [68]. The authors compared the efficiency of both constructs and showed that the pertuzumab-based construct was efficient at lower doses, but both induced potent tumor cell killing in vitro and reduced tumor growth in vivo. Interestingly the 2 antibodies used together seemed to synergize on Her2^med^ cell line LS174T. An anti-Glypican 3 (GPC3) × anti-CD16 antibody was developed using the same S-Fab format. GPC3 is a tumor antigen overexpressed in hepatocellular carcinomas (HCC) with low normal tissue expression. In this video article, the authors described the process of production of this antibody and showed an effective in vitro cytotoxicity effect on HCC cell lines [69].

### 3.2. Nanobody Coupling to An Antibody Recruiting Domain

Alternatively, recruiting NK cells on target cells and trigger ADCC can be achieved by the coupling of an antibody recruiting domain (ARD) to an antigen binding domain (ABD). One study used an anti-Her2 nanobody as ABD and a dinitrophenol group (DNP) as the ARD [70]. DNP is an environmental chemical contaminant, and around 0.8% of IgG1s circulating in human serum bind DNP. The ABD binds HER2 positive tumor cells, thereby recruiting DNP-specific IgG1 serum antibodies, which trigger NK cells through FcγRIII, resulting in tumor cell killing by ADCC. The modularity of this approach might translate into broad and potent therapies, however patient-to-patient variability in serum reactiveness to DNP could be an issue.

## 4. Modulation of Antigen Presenting Cells

Antigen presenting cells (APC) are key mediators of the adaptive immune response. APC phenotypes are very heterogeneous. M2 macrophages, myeloid derived suppressor cells (MDSCs) and tolerogenic dendritic cells promote an immunosuppressive environment and favor tumor growth and angiogenesis [71]. At the opposite of the spectrum, M1 Macrophages, B cells, and cDC1 dendritic cells mediate the anti-tumor immune response through antigen presentation [72,73]. These last populations are very important to trigger an effective lymphocyte response. Nanobody-based therapeutics can be used to reprogram or eliminate suppressor cells or to enhance anti-tumor APC effector functions.

### 4.1. Innate Immune Checkpoint Blockade

The SIRPα-CD47 axis is one of the most important innate checkpoints. CD47 is a ubiquitous marker of self and acts as a “don’t eat me” signal. It prevents phagocytosis by macrophages through interaction with the immune checkpoint receptor SIRP-α. By overexpressing CD47, many cancer types hijack this pathway to protect themselves from immune attacks [74]. Consequently, anti-CD47 nanobodies have been developed to restore macrophage-mediated phagocytosis. For instance, an anti-mouse CD47 nanobody significantly increased antibody-dependent phagocytosis of tumor cells by macrophages in vitro. However in vivo anti-tumor activity was only achieved in combination with anti-PD-L1 treatment [75]. The main issues with CD47 blockade are healthy cells toxicity (anemia) and antigen sink due to the ubiquitous expression of CD47. The challenge of CD47 therapy is thus to address the antibodies to the tumor site to reduce toxicity. Ingram et al. genetically engineered a B16 cell line to express the anti-CD47 nanobody within the tumor microenvironment to simultaneously address the antigen sink and nanobody intrinsic low half-life. This approach showed an effect of local CD47 therapy in combination with anti-TAA mAbs [76].

### 4.2. Nanobody-Based Immunization Strategies

One of the challenges in immunotherapy is to engineer dendritic cells to present TAA and provide effective immunization against the tumor. Adoptive transfer of autologous DCs matured in vitro to present TAA has been extensively studied in phaseI/II trials with promising results for glioma, melanoma, and lung cancers [77,78]. However, feasibility and cost of such strategies can negatively impact their therapeutic development. An easier and cheaper strategy for DC-based vaccine is to target TAA or TAA-derived peptides to the DC in vivo directly into patients. The major interests of nanobodies are their high penetrability within tissues and efficient coupling. Duarte et al. isolated nanobodies against the APC markers CD11b, CD36, and MHC-II combined to immunogenic peptides to determine the best marker for targeted antigen delivery [79]. An anti-MHC-II nanobody fused to an antigen was able to deliver the antigen to all DC and B cells subpopulations and induced a strong B cell response, as measured by serum responsiveness against the antigen. Interestingly, dimerization of the nanobody further increased B cell response, suggesting a role of antibody affinity or receptor clustering, or both, for internalization of the construction. This MHC-II nanobody was used to induce an immune response against the tumor antigen MUC1 [80]. Injection of the nanobody conjugated to MUC1-derived peptide induced strong humoral and CD4 responses. This strategy was applied to a different peptide format. A plant peptide scaffold was used to generate cyclic peptide and graft them onto the anti-MHC-II nanobody. Cyclization makes peptides more stable and less susceptible to proteolytic degradation. The nanobody targeting effectively activated an immunization against cyclical conformation of the peptides [81]. These studies highlight the potential of nanobody-conjugated TAA to induce an effective immune response against different antigen configurations in a non-invasive and cost-effective manner.

### 4.3. Drug Delivery

Macrophage mannose receptor (MMR)-expressing tumor associated macrophages (TAM) are involved in immune suppression and angiogenesis and are a marker of bad prognosis [82,83]. Specific elimination of the MMR^+^ macrophages is proposed as a therapeutic strategy to increase the M1/M2 ratio towards a more favorable for tumor regression. Nuhn et al. used a nanobody with a high affinity to MMR (~20 nM) and conjugated it to a polymeric nanogel to produce MMR-specific 40 nm particles carrying drugs or imaging agents [84]. In a mouse lung cancer model, they successfully induced fluorescent nanogel internalization by MMR^+^ TAMs and that phenotype was reverted in MMR KO mice. Drug delivery can be achieved by linking an internalization receptor. A nanobody-drug conjugate Targeting MHC-II on B cell lymphoma models presented a quick internalization and efficient drug delivery [85]. While this shows the potential of monovalent nanobody for MHC-II-dependent internalization and drug delivery, possibly representing a good alternative for therapy of anti-CD20 resistant lymphomas, it cannot be effectively use for specific APC targeting as it will kill pro-tumor as well as anti-tumor APCs.

### 4.4. APC Reprogramming

APC reprogramming from immunosuppressive to inflammatory phenotype has achieved great success in re-establishing an anti-tumor microenvironment in pre-clinical studies [86]. Gefitinib (Gef) and simvastatin (SV) are anti-tumor drugs with an effect on TAM activation [87,88]. In vitro treatment of macrophages with Gef/SV treatment inhibits HIF-2α and vascular endothelial growth factor expression. Recently, Yin et al. generated anti-PD-L1-nanobody-decorated liposomes to deliver Gef/SV treatment to PD-L1-expressing cancer cells and macrophages [89]. The liposome was effectively delivered to NSCLC endothelial cells and M2-like macrophages and the drug combination had potent anti-tumor effect on Gef-resistant and SV-resistant cells. Moreover, the M1/M2 ratio switched from M2-dominant to M1-dominant. This research shows the effective use of a nanobody to target both immune and cancer cells and remodel the tumor environment.

CD1d is a non-classical MHC involved in glycolipid presentation to NKT cells. This interaction activates NKT cell to produce cytokines and enhances IL-12 secretion and stimulatory capabilities of DCs. CD1d engagement potentiates anti-tumor T cell response [90]. Lameris et al. generated 22 anti-CD1d nanobodies with distinct biological activities [91]. Two of these nanobodies were able to induce NKT-independent IL-12 production by monocyte-derived DCs. Other nanobodies could block the NKT TCR-CD1d interaction or induce CD1d^+^ cell apoptosis, with a potential use for CD1d^+^ B lymphoblasts or multiple myelomas. This work highlights the small size of nanobodies and their ability to bind non-conventional epitopes as strong assets for the generation of molecules with various and well-defined effects.

Goyvaerts et al. developed a very interesting nanobody-lentivirus approach to address lentiviral vectors to specific cell populations [92]. The modified virus could effectively transduce DCs in a nanobody-dependent manner by targeting a so far unidentified target, with a strong potential for DC immunization. Unfortunately, the approach induced a weaker antigen-specific CD8^+^ response compared to a broad tropisms lentivirus, possibly due to an infection-related inflammation [93]. However, this works clearly demonstrates the potential of nanobody–lentivirus constructs to transduce cell populations for reprogramming and activation of dendritic cells.

## 5. Targeting the Tumor Environment Cytokines and Chemokines

### 5.1. Pro-Tumor Cytokines Targeting: TNF/G-CSF

Cytokines are key modulators of immune cell states of activation. Inflammatory cytokines and growth factor are involved in cancer progression. Another immunotherapy approach aims at modulating the immune cells or cancer cells activity by targeting cytokines and their receptors.

TNF-α is a pro-inflammatory cytokine inducing many cellular processes, among which cell death, but also proliferation and angiogenesis. TNF-α serum levels are increased in cancer patients and correlate with disease progression [94]. Many tumors evolve to escape TNF-α-mediated cytotoxicity and use it as a growth factor helping their survival and migration. Moreover TNF-α acts as an immunosuppressive cytokine by increasing MDSC and Treg proliferation [95,96]. Anti-TNF-α blockade has shown interesting anti-tumor effects [97]. An anti-TNF-α nanobody was able to reduce TNF-mediated proliferation and migration potency of breast cancer cell lines. The nanobody alone did not significantly reduce tumor growth in vivo but greatly inhibited lung metastasis and increased potency of the antimitotic Paclitaxel [98].

An alternative to block a cytokine activity is to target its receptor expressed on tumor cells. Bakherad et al. isolated an anti-granulocyte-colony stimulating factor receptor (G-CSFR) nanobody [99]. G-CSFR is mostly expressed in neutrophils and mediates their proliferation and activity. G-CSFR is expressed on gastric, colon, or lung carcinomas and leads to increased tumor growth and metastasis in a G-CSF-dependent matter [100,101,102]. In this study, the blocking nanobody successfully inhibited the G-CSF-induced gastric cancer cell proliferation in vitro via the SOCS3 signaling pathway.

### 5.2. Modulating the CXCR4/CXCR7/CXCL12 Chemokines Axis in Solid Cancers

Chemokines are important signals to orient cell migration within the organism, in particular for the immune cell infiltration into the tumor [103]. However, cancer cells can also use these signals to favor their migration into the blood stream. The CXCR4/CXCR7/CXCL12 chemokine axis is physiologically critical for hematopoiesis and T cell homing into inflammation sites. Chemokines CXCR4 and CXCR7 are overexpressed in many solid cancers and involved in metastasis towards CXCL12-rich tissues (such as lung, liver, and bone marrow) [104,105]. CXCR7 can also induce angiogenesis and tumor growth independently of its effect on tumor cell migration. CXCR4 blocking strategies using antibodies [106] or small inhibitors [107,108] have proven to be effective to reduce metastatic burdens and a phase IIb clinical trial for a CXCR4 antagonist in combination with anti-PD1 therapy in advanced pancreatic cancer is ongoing (NCT02907099).

Some nanobodies targeting immune cell chemotaxis are being developed to prevent tumor cell migration. An anti-CXCR7 nanobody was selected to block its interaction with CXCL12. The nanobody reduced tumor growth via its anti-angiogenesis effect [109]. However, blocking CXCR7 alone does not appear sufficient to stop epithelial-mesenchymal transition and metastasis. It would thus be of interest to associate CXCR7 and CXCR4 blockade to reduce CXCL12-mediated migration in addition of the anti-tumor effect of CXCR7 antagonists [110,111]. Several blocking anti-CXCR4 nanobodies have been used to reduce T cell chemotaxis in HIV infections contexts. These nanobodies prevented CXCL12-mediated migration of CXCR4^+^ cells [112,113]. By fusing these nanobodies to an Fc domain, the authors increased the apparent affinity and the blocking potency against CXCR4, and induced ADCC and complement dependent cytotoxicity of CXCR4-expressing cells [114]. To our knowledge these anti-CXCR4 nanobodies have not been tested in cancer cell migration models but the combination of anti-CXCR4 and CXCR7 blocking nanobodies could be synergizing tools to tackle CXCL12-mediated metastasis.

### 5.3. Nanobody as Carriers for Cytokine Delivery

With the well-demonstrated anti-tumor effect of some cytokines, numerous anti-tumor cytokines have been proposed as cancer treatment. IL-2, a cytokine involved in immune cell activation and proliferation, was the first immunotherapy approved for cancers as early as 1992 [115]. However, while cytokines have great anti-tumor potency, the main limitation is off-target activation and associated toxicity. The main strategies to circumvent this limitation are gene transfer to produce cytokines locally, or antibody-directed cytokines (immunocytokines). Nanobodies can also be used as carrier for cytokines to restrict the activation to the tumor microenvironment. Dougan et al. used an anti-PD-L1 nanobody to deliver the anti-tumor cytokines IL-2 and IFN-γ to immunologically impaired pancreatic tumors [116]. The nanobody was able to deliver the cytokines in well vascularized B16 melanoma tumors but also to denser mice pancreatic tumors, whilst the nanobody-Fc fusion did not achieve equally well. Both IL-2-nanobody and IFN-γ-nanobody synergized with an anti-TAA antibody for tumor growth inhibition. Immune infiltrate analysis showed increased CD8^+^ counts but also Tregs proliferation with the IL-2 construct. The IFN-γ-nanobody significantly decreased MDSCs and redirected the DCs towards a MHC-II^+^ phenotype. A similar approach was developed by Liu et al. with an IL15-Linker-IL-15Rα construct fused to the C-terminus of an anti-CEA nanobody-Fc fusion [117]. IL-15 binding to its soluble receptor IL15-Rα increases IL-15 effector function [118]. Contrarily to the broad spectrum of IL-2, IL-15 mostly affects NK and CD8^+^ T cells proliferation and activation. Despite the relatively large size of this construct (~140 kDa), the authors demonstrated a strong anti-tumor effect associated with CD8^+^ recruitment within the tumor in mouse models.

## 6. Nanobodies as Potent Imaging Tools

### 6.1. Importance of Molecular Imaging for Cancer Diagnostics

**Figure 3:**
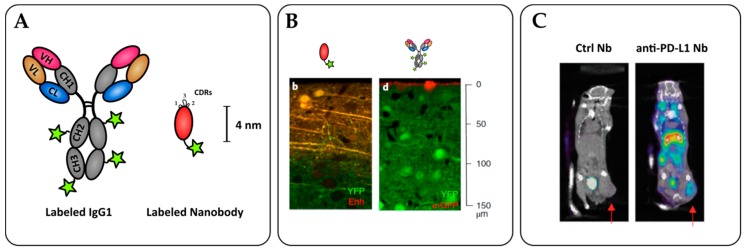
Nanobodies as potent tools for tumor immunoimaging. (**A**) Nanobody labelling strategies allow for site-specific and oriented conjugation. (**B**) Penetration of an anti-GFP nanobody (left) or full-size IgG (right) within an YFP-expressing brain tissue in vitro. Adapted from Fang T. et al. [120]. (C) SPECT/CT imaging of PD-L1 positive mouse lung epithelial cell line TC-1 in C57/BL6 mice with radiolabeled ^99m^Tc nanobodies 1 h after injection. The arrows indicate the tumor site. Adapted from Broos K. et al. [124].

With the ongoing development of in targeted therapies, it has become more and more important to visualize the presence tumor antigens and immune infiltrates to predict responsiveness. Molecular imaging with labeled antibodies has been intensely developed but the difficult tissue penetration and long half-life are strong obstacles to obtain good contrast and cancer detection. Consequently, nanobodies recently emerged as powerful tools for in vivo and in vitro imaging for diagnosis [119]. As opposed to the therapeutic setting, the fast elimination of nanobodies in vivo due to their small size and the absence of Fc fragment avoiding recycling constitutes a strong advantage for imaging, as it reduces background and generates a high contrast rapidly after injection. For in vitro staining, the small size of nanobodies also affords a better tissue penetration and staining compared to full size IgG (Figure 3) [120]. Nanobody-based imaging agents are very promising for an accurate and fast diagnosis in cancer therapy. Immuno-imaging requires the labeling of the targeting agent with an imaging probe. NHS ester-based chemistry targeting lysine side chains is often not ideal for nanobody labeling, because their small size is associated with a relatively high risk of impacting their binding activity. Moreover, it is of crucial importance to control batch-to-batch reproducibility concerning labeling ratio and orientation. Most current strategies are using sortase-mediated coupling. Sortase is a transpeptidase coupling a LPXTG motive to an N-terminal glycine [121,122]. This strategy is a very simple, cost-effective, and versatile way to label biomolecules in a controlled fashion. Moreover, the reaction cleaves the end of the LPXTG motive, allowing the convenient use of a purification tag (such as polyHis), which is ultimately replaced by the probe after coupling. Other site-directed coupling strategies can be used, such as His tag directed coupling using ^99m^Tc-tricarbonyl for Single-photon emission computed tomography (SPECT) [123].

### 6.2. Cancer Cell Detection

The most advanced molecule in non-invasive imaging is an anti-Her2 nanobody used to detect Her2 expression in breast cancers via positive electron tomography-computed tomography (PET/CT). A phase I trial demonstrated a quick elimination, allowing measurement at 60–90 min after injection [125]. An ongoing phase II clinical trial investigates the uptake of the radiopharmaceutical 68-GaNOTA-Anti-HER2 nanobody in brain metastasis using PET/CT imaging (NCT03331601). Many nanobodies, including anti-CD20 [126], anti-Dipeptidyl-Peptidase 6 [127], and anti-HER3 nanobodies [128] are currently being studied.

### 6.3. Monitoring of Immune Infiltration

Nanobody-based imaging agents are very promising for an accurate and fast diagnosis in cancer therapy. Importantly, nanobodies are been explored as imaging agents to assess the immune infiltration within tumors prior or during immunotherapy. Indeed, therapeutic responses and patient prognosis are often linked to the nature, density, and activation status of immune cells infiltrated within the tumor microenvironment. Understanding the immune contexture of tumor would help in delivering the right treatment to patients.

#### 6.3.1. Tumor Infiltrating Lymphocyte Monitoring

T cells are a particularly interesting target for molecular imaging as their infiltration and state of activation are crucial to immunotherapy responsiveness. Bannas et al. used an anti-ART2 nanobody as imaging tool for optical imaging of T cells within lymph nodes. While a nanobody-Fc fusion displayed better in vitro staining, the single domain nanobody had more imaging potency in vivo due to reduced background. Good signal/noise ratio was observed 2 h post administration, while a monoclonal antibody required at least 24 h. Interestingly, this nanobody possesses a functional ART2 inhibiting property and could be used for both therapy and immunomodulation [129]. A PEGylated ^89^Zr anti-CD8^+^ nanobody was produced to monitor the CD8^+^ T cell infiltration within the tumor by PET/CT. PEGylation with a 20 kDa PEG moiety greatly reduced kidney retention of the antibody and thus gave a more specific staining of lymphoid organs [130]. In an immunized B16 mouse model giving partial regression with anti-CTLA-4 therapy, the authors showed that a homogeneous CD8^+^ infiltration detected with the anti-CD8 nanobody in PET/CT correlated with anti-CTLA-4 responsiveness. Direct assessments of the checkpoint blockade targets have been investigated with a ^99m^Tc-laballed anti-PD-L1 nanobody for SPECT imaging. Potent PD-L1^+^ tumor labeling was achieved 1 h post-inoculation and PD-L1 expression correlated with CD8^+^-dependent reduced tumor development [124].

#### 6.3.2. APC Monitoring

Macrophage polarization is another important prognosis marker. The ratio between pro and anti-inflammatory macrophages shapes the tumor microenvironment.

Anti-MMR nanobodies have been evaluated to detect pro-tumor TAMs. Using a ^99m^Tc-labeled nanobody, Movahedi et al. managed to detect TAMs in mice [131]. In a following study, the authors compared the use of an anti-MMR nanobody with ^99m^Tc or ^18^F labeling respectively for SPECT or PET/CT imaging. Their work showed a greatly reduced liver and kidney uptake of the fluorinated nanobody compared to the radiometal-labeled version [132].

Conversely to MMR, MHC-II is a good prognosis marker and is associated with an efficient antigen presentation. An anti-MHC-II nanobody labeled with a near infrared fluorochrome was used to monitor human cell infiltration in NOD/SCID humanized mice. That probe was efficient for organ labeling and flow cytometry analysis, but the authors used a ^64^Cu staining for in vivo imaging. The nanobody displayed a good PET/CT signal/noise ratio 2 h after injection, and allowed imaging of MHC-II^+^ cells in the spleen and bones despite kidney and bladder non-specific accumulation [133]. To take advantage of the higher availability of ^18^F-2-deoxyfluoroglucose (^18^F-FDG) compared to other ^18^F probes, the authors developed an interesting strategy using ^18^F-FDG as a labeling probe. The labeled high affinity nanobody could detect MHC-II lymphoid organs more efficiently than the previously described low affinity anti-MHC-II ^18^F-nanobody. This approach allowed the detection of early tumors (~1 mm diameter) that cannot be detected with ^18^F-FDG due to their low metabolic activity [134]. This strategy combines the feasibility of ^18^F-FDG PET/CT imaging and precision of molecular nanobody-based imaging.

## 7. Conclusions

As shown by the multitude of different formats with promising pre-clinical effects reported in the last years, nanobodies emerge as powerful antibody engineering tools to replace scFv fragments as building blocks, and allow the generation of molecules with carefully designed affinities, valencies, and specificities. Nanobodies also rise as very potent imaging agents due to their stability, production and coupling efficiency, tissue penetrability, and fast elimination from the blood stream. We forecast that, in the near future, nanobodies will lead to many innovative and high potential molecules for cancer immunoimaging and immunotherapy.
